# The impact of social determinants of health on outcomes of brexucabtagene autoleucel in adults with relapsed/refractory B-cell acute lymphoblastic leukemia

**DOI:** 10.1038/s41409-025-02693-0

**Published:** 2025-08-23

**Authors:** Timothy E. O’Connor, Chenyu Lin, Gregory W. Roloff, Amy Zhang, Katharine Miller, Ibrahim Aldoss, Noam E. Kopmar, Simone E. Dekker, Vishal K. Gupta, Nikeshan Jeyakumar, Ibrahim N. Muhsen, Yannis Valtis, Naveed Ahmed, Katherine Sutherland, Kaitlyn C. Dykes, Mohamed Ahmed, Evan Chen, Hector Zambrano, Danielle Bradshaw, Santiago Mercadal, Marc Schwartz, Sean Tracy, Matthew P. Connor, Michal Kubiak, Akash Mukherjee, Navneet Majhail, Minoo Battiwalla, Luke Mountjoy, Shahbaz A. Malik, John Mathews, Paul Shaughnessy, Betsy Blunk, Aaron C. Logan, Abdullah Ladha, Anjali S. Advani, Maryann Stefan, Caitlin Guzowski, Rasmus T. Hoeg, Talal Hilal, Jozal Moore, Kristen M. O’Dwyer, LaQuisa C. Hill, Joshua Sasine, Caspian Oliai, Melhem M. Solh, Catherine J. Lee, Vamsi K. Kota, Divya Koura, Muthu V. Kumaran, Jessica T. Leonard, Noelle V. Frey, Jae H. Park, Marlise R. Luskin, Veronika Bachanova, Ahmed Galal, Vinod Pullarkat, Michael R. Bishop, Wendy Stock, Ryan D. Cassaday, Bijal D. Shah, Rawan Faramand, Lori S. Muffly, Stephanie B. Tsai, Bhagirathbhai Dholaria

**Affiliations:** 1 Loyola University Medical Center, Maywood, IL USA; 2 Duke University, Durham, NC USA; 3 University of Chicago, Chicago, IL USA; 4 Stanford University School of Medicine, Stanford, CA USA; 5 City of Hope National Medical Center, Duarte, CA USA; 6 Fred Hutchinson Cancer Center & University of Washington School of Medicine, Seattle, WA USA; 7 University of California Los Angeles, Los Angeles, CA USA; 8 Center for Cell and Gene Therapy, Baylor College of Medicine, Houston, TX USA; 9 Memorial Sloan Kettering Cancer Institute, New York City, NY USA; 10 University of California San Diego, La Jolla, CA USA; 11 Cedars-Sinai Medical Center, Los Angeles, CA USA; 12 Dana Farber Cancer Institute – Harvard Medical School, Boston, MA USA; 13 Sarah Cannon Transplant and Cellular Therapy Program at TriStar Centennial Medical Center – HCT Healthcare, Nashville, TN USA; 14 Augusta University – Georgia Cancer Center, Augusta, GA USA; 15 University of Utah Huntsman Cancer Institute, Salt Lake City, UT USA; 16 University of Colorado, Aurora, CO USA; 17 University of Minnesota, Minneapolis, MN USA; 18 University of Pennsylvania, Philadelphia, PA USA; 19 Billings Clinic, Billings, MT USA; 20 University of Arkansas for Medical Sciences, Little Rock, AR USA; 21 Sarah Cannon Cancer Institute, Nashville, TN USA; 22 Colorado Blood Cancer Institute, Denver, CO USA; 23 Texas Transplant Institute, Austin, TX USA; 24 Sarah Cannon Transplant and Cellular Therapy Program, Dallas, TX USA; 25 Sarah Cannon Transplant and Cellular Therapy Program at Methodist Hospital, San Antonio, TX USA; 26 University of California San Francisco, San Francisco, CA USA; 27 University of Southern California, Los Angeles, CA USA; 28 Cleveland Clinic, Cleveland, OH USA; 29 Northside Hospital Cancer Institute, Atlanta, GA USA; 30 University of California Davis, Davis, CA USA; 31 Mayo Clinic Arizona, Phoenix, AZ USA; 32 University of Rochester, Rochester, NY USA; 33 Oregon Health & Science University, Portland, OR USA; 34 H. Lee Moffitt Cancer Center, Tampa, FL USA; 35 Vanderbilt University Medical Center, Nashville, TN USA

**Keywords:** Acute lymphocytic leukaemia, Immunotherapy

## Abstract

Brexucabtagene autoleucel (brexu-cel) is a chimeric antigen receptor T (CAR T) cell therapy approved for adults with relapsed or refractory (R/R) B-cell acute lymphoblastic leukemia (B-ALL). We studied the impact of social determinants of health (SDoH) on outcomes of adults with B-ALL receiving brexu-cel. This retrospective analysis included adults (≥18 years) with R/R B-ALL treated with brexu-cel between 2021 and 2023. Cox proportional hazards models evaluated the association of race, ethnicity, and SDoH with progression-free survival (PFS) and overall survival (OS). 189 patients received brexu-cel and 57% were male. 55% were non-Hispanic White, 30% Hispanic, 7% non-Hispanic Black, 6% Asian/Pacific Islander, and 2% other/unknown. 43% were referred from private/community-based practices and 35% lived 50 miles or greater from the CAR T center. Health insurance included public (47%) and private (41%). 31% had a high social deprivation index (SDI, 76–99th percentile). Black race was associated with worse OS (HR 3.48; 95% CI 1.01–12.03). There was no difference in PFS (HR 1.03, 95% CI 0.50–2.10) or OS (HR 1.43; 95% CI 0.56–3.65) in Hispanic patients. Outcomes appear independent of SDoH and SDoH did not impact OS. We observed comparable outcomes to non-Hispanic patients.

## Introduction

B-cell acute lymphoblastic leukemia (B-ALL) is a rare hematologic malignancy that accounts for 0.3% of all new cancer diagnoses in the U.S., with an estimated 6500 combined new pediatric and adult cases expected for 2024 [1]. While B-ALL is more commonly seen in children, the majority of ALL-related deaths occur in the adult population [1]. The development of novel immunotherapies including blinatumomab and inotuzumab ozogamicin allows a greater number of adults with relapsed or refractory (R/R) B-ALL to achieve complete response (CR), but these agents rarely result in durable remissions [2, 3].

Brexucabtagene autoleucel (brexu-cel) is an autologous CD19-directed chimeric antigen receptor T (CAR T) cell therapy that received U.S. Food and Drug Administration (FDA) approval in October 2021 as the first CAR T cell therapy for adults older than 25 years with R/R B-ALL, and the second product approved for adolescent young adults between 18 and 25 years [4]. It was subsequently approved by the European Commission (EC) in 2022 for the treatment of adults aged 26 years and above with R/R B-ALL. Approval was based on the results of the ZUMA-3 trial, in which 71% of patients achieved a CR with a median relapse free survival of 11.6 months and median overall survival (OS) of 18.2 months [5]. With extended median follow-up of 41.6 months, the median OS (95% CI) was 25.6 months (1.2–47.0; *N* = 78) and was 38.9 months (25.4-not estimatable) for responders (*N* = 58) [6].

It has become increasingly recognized that racial and ethnic disparity is pervasive in many diseases and cancers. In particular, B-ALL has a higher incidence rate in Hispanic patients compared to other ethnic groups [7, 8]. Data from the Surveillance, Epidemiology, and End Results (SEER) database suggest that Black race and Hispanic ethnicity are associated with inferior survival in B-ALL [9, 10]. In Hispanic patients, these findings may be partially explained by an increased incidence of Philadelphia chromosome (Ph)-like B-ALL [11, 12], which is associated with treatment failure, relapse, and inferior survival [13, 14]. By contrast, survival outcomes for Black patients have been found to be worse, even when adjusting for overall disease risk [15]. Social determinants of health (SDoH), such as insurance status, access to care, and income, have also been found to impact survival in B-ALL [9, 16].

Novel strategies are continuously sought to close these historic disparities in outcomes. Using data from the Real-world Outcomes Collaborative of CAR T in Adult ALL (ROCCA) consortium, this study aimed to assess for inequities in clinical outcomes due to race, ethnicity, and SDoH in adult patients with R/R B-ALL infused with brexu-cel.

## Methods

ROCCA is comprised of 31 U.S. academic and community-based centers and was created to study adults with R/R B-ALL who received CAR T cell therapies outside a clinical trial setting. Adult patients (≥18 years old) with R/R B-ALL who received commercial brexu-cel between October 2021 and August 2023 were included in this analysis. Data were collected retrospectively, and the data lock for this analysis occurred on October 30, 2023. Patients who received brexu-cel as part of a clinical trial or an expanded access protocol prior to FDA approval were excluded.

The study was approved by Institutional review board (IRB) and ethics committee at Stanford University. IRB approval was granted at each participating center. All methods were performed in accordance with the relevant guidelines and regulations. Informed consent was obtained for CAR T therapy from all participants. Data were entered into a HIPAA-compliant database (REDCap^®^) housed at Stanford University.

The purpose of this study was to assess if and how race, ethnicity, and certain SDoH (referral source, insurance, distance from home to CAR T site, and the social deprivation index) correlated with progression-free survival (PFS) and overall survival (OS) after receipt of brexu-cel. Race and ethnicity were self-reported, per chart review. The social deprivation index (SDI) is a composite measure (including income, education, employment, housing, household characteristics, and transportation) used to quantify socio-economic variation in health outcomes, with a higher SDI indicating greater social disadvantage [17]. For our patient population, the SDI was estimated at the zip code level and patients were divided into quartiles based on their SDI score: low (0–25th percentile), low-medium (26–50th percentile), medium-high (51–75th percentile), and high (76–99th percentile). Household income was not included in the analysis due to missing data and possibility of collinearity with SDI. Fifty miles to the CAR T treating site was chosen as the distance threshold for likelihood of patients requiring local lodging.

PFS was measured from the time of brexu-cel infusion to either disease relapse, non-response, or death from any cause, and censored at last follow-up. Overall survival was measured from the time of brexu-cel infusion to death from any cause, censored at last follow up Non-relapse mortality was measured using Gray’s test, with relapse considered a competing risk. Univariable and multivariable Cox proportional hazards models were used to evaluate the association of race, ethnicity, age, sex, pre-apheresis disease burden, and SDoH (referral source, insurance type, distance from home to CAR T site, and the SDI) with PFS and OS. The variables used were pre-specified without a p-value cutoff, and patients with missing preselected variables were removed from the model. Kaplan–Meier unadjusted curves for PFS and OS were constructed with censoring at the time of last follow-up. A chi-squared test was used to compare toxicity outcomes. Analysis was conducted using SAS Version 9.4.

## Results

### Patient characteristics and social determinants of health

The analytic cohort consisted of 189 adult patients who were infused with brexu-cel. Baseline descriptive characteristics can be found in Table 1. Most patients (71%) were between ages 18 and 59 years, and more than half (57%) were male. In total, 104 (55%) patients identified as non-Hispanic white, 56 (30%) as Hispanic, 13 (7%) as Black, 12 (6%) as Asian/Pacific Islander, and 4 (2%) as other or were unknown. Patients were most commonly referred from community/private practice oncology settings (43%) or academic oncology practices (41%). All patients were reported to have health insurance coverage, with most having either public (47%) or private (41%) health insurance. Many patients (34%) relocated for CAR T therapy. Thirty-five percent lived 50 miles or greater from the CAR T site. Patients came from all socioeconomic backgrounds with 39 (21%) having a low SDI, 38 (20%) a low-medium SDI, 32 (17%) a medium-high SDI, and 58 (31%) a high SDI. The SDI was unknown for 22 (12%) patients.

**Table 1:** Baseline clinical characteristics, demographic characteristics, and social determinants of health.

	*N (%)*
Number of patients	*189*
Sex	
Female	82 (43)
Male	107 (57)
Race/Ethnicity	
Non-Hispanic white	104 (55)
Hispanic	56 (30)
Black	13 (7)
Asian/Pacific Islander	12 (6)
Other	3 (2)
Unknown	1 (1)
Age at CAR T infusion, years	
18–59	135 (71)
60+	53 (28)
Unknown	1 (1)
Referral source	
Private/Community practice	82 (43)
Government/Public	5 (3)
Academic institution	77 (41)
Other	3 (2)
Unknown	22 (12)
Insurance	
Government/Public	89 (47)
Private	77 (41)
Other	6 (3)
Unknown	17 (9)
Distance to CAR T site	
Less than 50 miles	110 (58)
50 miles or greater	67 (35)
Unknown	12 (6)
Relocated for CAR T therapy	
Yes	64 (34)
No	109 (58)
Unknown	16 (8)
Social deprivation index	
Low (0–25th percentile)	39 (21)
Low-medium (26–50th percentile)	38 (20)
Medium-high (51–75th percentile)	32 (17)
High (76–100th percentile)	58 (31)
Unknown	22 (12)
Disease subtype	
Ph+	55 (29)
Ph-like	34 (18)
Ph-negative	100 (53)
Pre-apheresis disease status	
Active disease at time of apheresis	94 (53)
CR, MRD+	47 (26)
CR, MRD-	28 (16)
CR, achieved by morphology without MRD testing	4 (2)
Unknown	16 (8)
Pre-apheresis bone marrow blast %	
<25%	100 (68)
25–74%	33 (22)
75%	14 (10)
Missing / not performed	42 (22)
Extramedullary disease at apheresis	
Yes	43 (23)
No/Unknown	146 (77)
CNS involvement at apheresis	
Yes	34 (18)
No/Unknown	155 (82)
Number of prior lines of therapy, median (range)	4 (2–12)
Prior blinatumomab therapy	112 (59)
Prior inotuzumab ozogamicin therapy	90 (48)
Prior HSCT	78 (41)

*CAR T* chimeric antigen receptor T-cell, *Ph* Philadelphia chromosome, *CR* complete response, *MRD* measurable residual disease, *CNS* central nervous system; HCST, hematopoietic stem cell transplant.

### Safety and toxicity

There were no significant differences in the rates of high-grade toxicity (grade ≥3 cytokine release syndrome or immune effector cell associated neurotoxicity syndrome) when comparing non-Hispanic white to Hispanic patients (*p* = 0.50), public to private insurance (*p* = 0.26), less than 50 miles to greater than 50 miles from the CAR T site (*p* = 0.46), and low SDI (0–25th percentile) to high SDI (76–100th percentile) (*p* = 0.48) (Table 2).

**Table 2:** High grade toxicity across race and social determinants of health.

	*Grade* ≥ *3 CRS or ICANS*	*Grade* < *3 toxicity CRS or ICANS*	*p*-value
Race/Ethnicity			0.50
Non-Hispanic white	39 (38)	65 (62)	
Hispanic	18 (32)	38 (68)	
Insurance			0.26
Government/Public	34 (39)	54 (61)	
Private	23 (30)	53 (70)	
Distance to CAR T site			0.46
Less than 50 miles	36 (33)	72 (67)	
50 miles or greater	26 (39)	41 (61)	
Social deprivation index			0.48
Low (<25th percentile)	11 (28)	28 (72)	
High (>75th percentile)	20 (35)	37 (65)	

*CRS* cytokine release syndrome, *ICANS* immune effector cell-associated neurotoxicity syndrome, *CAR T* chimeric antigen receptor T-cell.

### Response and survival

There was no significant difference in response rates (complete response/measurable residual disease negative, complete response/measurable residual disease positive, or unknown) comparing Hispanic (OR 0.06; 95% CI 0.00–0.86), Black (OR 0.03; 95% CI 0.00–0.57), or Asian/Pacific Islander (OR 0.11; 95% CI 0.00–2.57) patients to non-Hispanic white patients. There was also no difference in the likelihood of response based on insurance type (OR 2.65; 95% CI 0.47–15.01) or distance to the CAR T site (OR 2.93; 95% CI 0.58–14.73). There was likewise no difference in response rate when comparing low SDI to high SDI (OR 0.24; 95% CI 0.02–2.58) (Supplementary Table 1).

**Fig. 1:**
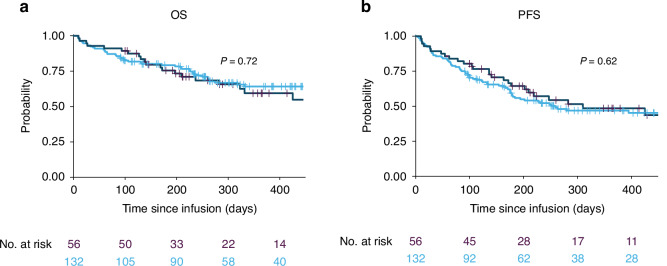
Outcomes of Hispanic patients compared to non-Hispanic patients after brexu-cel for B ALL. **a** overall survival. **b** Progression-free survival.

In univariable analyses, there were no differences in OS based on race, referral source, insurance status, or distance to CAR T site. (Supplementary Table 2) There was no difference in OS between Hispanic patients and non-Hispanic patients (HR 1.10; 95% CI 0.65–1.87) (Fig. 1a). Median OS was not reached. There was no difference in PFS between Hispanic patients and non-Hispanic patients (HR 0.89; 95% CI 0.57–1.40) (Fig. 1b). Median PFS was 310 days for Hispanic patients and 259 for non-Hispanic patients. There was no difference in PFS comparing low SDI (0–25th percentile) to the upper 3 SDI quartiles combined (26–99th percentile) (HR 1.67; 95% CI 0.94–2.97). No other SDoH factors were associated with worse PFS (Supplementary Table 2). Cumulative incidence of non-relapsed mortality (NRM) also did not differ by race *(*Black: HR 1.18, 95% CI 0.27–5.20; Hispanic: HR 0.91, 95% CI 0.37–2.24; Other: HR 0.99, 95% CI 0.23–4.23) or SDI (Low: HR 0.85, 95% CI 0.25–2.90; Low-medium: HR 1.41, 95% CI 0.47–4.19; Medium-high HR 1.37, 95% CI 0.45–4.22). (Supplementary Fig. 1a) (Supplementary Fig. 1b).

**Fig. 2:**
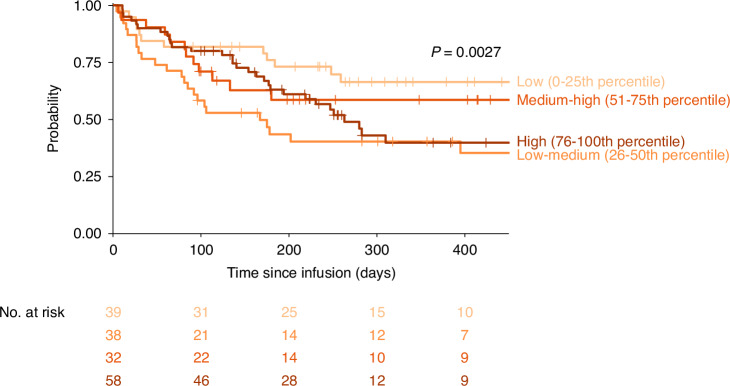
Progression free survival by social deprivation index quartiles.

In multivariable analysis, black race was associated with worse OS when compared with non-Hispanic white patients (HR 3.48; 95% CI 1.01–12.03) (Supplementary Table 3), whereas Hispanic (HR 1.43; 95% CI 0.56–3.65) and Asian/Pacific Islander (HR 1.62; 95% CI 0.34–7.72) patients did not have significantly worse OS when compared to non-Hispanic white patients. Distance to the CAR T site, insurance type, and SDI did not significantly impact OS. There was no difference in PFS when comparing Hispanic (HR 1.03; 95% CI 0.50–2.10) and Black (HR 1.90; 95% CI 0.73–4.94) to non-Hispanic white patients, government/public to private insurance (HR 1.01; 95% CI 0.56–1.82), or distance to the CAR T site (HR 1.37; 95% CI 0.72–2.60) (Supplementary Table 4). PFS did differ based on SDI in multivariable models (Fig. 2).

## Discussion

CAR T cell therapy has revolutionized the treatment of relapsed/refractory hematologic malignancies, including R/R B-ALL. In the pre-immunotherapy era, factors such as race, ethnicity, and SDoH have been shown to impact outcomes in B-ALL [18–21]. However, how they relate to and affect outcomes in patients with B-ALL receiving CAR T therapy is not well established. Large national cancer center databases currently lack the granularity required to clearly assess the delivery and impact of such novel therapies. Meanwhile, clinical trials often fail to reflect real-world demographics. Multicenter retrospective studies can provide more discernable variations in access, efficacy, and safety of novel therapies. In contrast to the ZUMA-3 trial, which included 67% non-Hispanic white, 20% Hispanic, and 2% non-Hispanic Black patients [5], our patient population was 55% non-Hispanic white, 30% Hispanic, and 7% non-Hispanic Black, which is more consistent with the real-world demographics of B-ALL patients across the US [8]. Population-based studies have suggested worse outcomes for Hispanic and Black patients with ALL [9, 22, 23], which are thought to be related to a variety of factors, including differences in mutational patterns and disease risk [11, 12], as well as socioeconomic status (SES) [24]. We show that Hispanic patients had similar outcomes compared to non-Hispanic white patients after receipt of brexu-cel. Worse OS was seen in multivariable analysis comparing Black patients to non-Hispanic white patients, which has been observed in studies of the anti-CD19 CAR T-cell therapy tisagenlecleucel [25], but not with axicabtagene ciloleucel [26]. We cautiously interpret these results, however, given the wide confidence interval and the small sample size of non-Hispanic Black patients. This difference in OS was not seen in univariable analyses, and there was no significant difference in PFS or NRM, making it difficult to provide an explanation for the observed marginally inferior OS in multivariate analyses only. Overall, it is likely the number of Black patients in our cohort is too few to definitively conclude worse survival following treatment with brexu-cel, but this issue merits further investigation with larger sample sizes and more experience. Our group previously demonstrated that response rates with brexu-cel are high, in line with data from ZUMA-3 [27]. We found no significant difference in response rates among any specific race or ethnicity, which contrasts with other CAR T studies [26, 28]. Comparisons of these studies’ findings to our patient cohort are limited due to differing focus in disease type and CAR T products. Overall, these results are promising and support that the historically poor outcomes of traditionally underrepresented US cancer patients may be mitigated by specialty cancer care and access to CAR T [29–31].

When investigating impact of insurance, we found that patients with public insurance were more likely to have a complete response (*p* = 0.03). However, this finding did not translate into differences in OS (*p* = 0.63) or PFS (*p* = 0.98). Our study contrasts with literature indicating that patients with public insurance have worse outcomes, possibly due to delays in care and a tendency to present with more advanced disease, which limit treatment options [16, 32, 33]. Additionally, other studies have found that patients with public insurance are less likely to receive CAR T therapy [34]. Interestingly, a large proportion of our cohort were publicly insured (47%), suggesting that insurance type, in and of itself, does not seem to be a primary barrier to receiving this therapy in the US. However, our patients include only those that made it to CAR T infusion, presumably with specialized support from social workers, case managers, and financial advisors embedded within the hospital system and cell therapy program. This finding emphasizes the importance of expanding access and improving affordability of CAR T-cell therapies through better collaboration among the healthcare system including referral base, payers, and pharmaceutical manufacturers. Greater emphasis should be placed on equity and quality-adjusted life-years gained in the context of a disease with a relatively poor prognosis [35]. Historically disadvantaged patients may fair just as well so long as they are given the opportunity to receive novel therapies [36].

We also captured distance to the CAR T site, an important variable reflecting a barrier to access to care and overall geographical distribution of race and SES [37]. Nearly half of patients in our cohort lived >50 miles from the CAR T cell center, necessitating relocation for this therapy. While we did not assess the associated financial toxicity of distance on the patient, caregiver, or healthcare system, others have shown that distance to the treatment center is strongly associated with likelihood of receiving CAR T [34]. However, other studies, which included patients who received CAR T, have shown that distance does not affect PFS or OS [38]. Likewise, we found that distance to the CAR T site had no impact on PFS (*p* = 0.34) or OS (*p* = 0.15). Requirements to be near tertiary centers after advanced therapies such as CAR T and stem cell transplant have long been a barrier to patients’ ability to receive them. CAR T manufacturers’ compensation to patients and family for local lodging, gasoline, and meals likely help offset the distance barrier. Increasing access to CAR T therapy in community centers and expanding treatment to the outpatient setting with the use of telemedicine and remote digital monitoring technology may allow for more patients to be treated closer to home, leading to greater equity in geographic allocation of therapy [39–42].

Patients from more disadvantaged communities may be less likely to receive CAR T [34] and more likely to have worse outcomes [43]. In order to approximate SES, we used the SDI which was estimated at the zip code level. This type of index was validated by Butler et al. based on census tracts at the county level in the United States, where they showed a positive relationship between high SDI and worse mortality that persisted when controlling for other measures of access to care [17]. Additionally, they found that the SDI was more predictive of a health outcome measure than other determinants of health, such as insurance status or ethnicity. The use of the SDI is particularly important in the United States because persons from areas with a higher index have limited access to care leading to poorer patient outcomes. Furthermore, a high SDI has been shown to correlate with increased hospitalization and death from COVID-19 [44], lower rates of colorectal cancer screening [45], and higher stage at diagnosis in breast cancer [46], strengthening its use and applicability in this setting. Our data suggest that in the real-world setting, patients with varying degrees of socioeconomic advantage are being referred for and are receiving brexu-cel; almost half (48%) of patients were from a neighborhood with a medium-high or high SDI, indicating a greater degree of social disadvantage [17]. However, our dataset did not capture the degree of those who did not make it to CAR therapy in the first place. We found no difference in OS based on SDI, which is consistent with other studies of CAR T in pediatric ALL [47]. Our finding underscores that access to this advanced treatment may be as important as various disease determinants or objective clinical data in determining outcomes. Other studies have shown that ethnicity may impact outcomes after allogeneic transplant in the UK [48]. This difference may reflect the more intricate post-transplant care required compared to CAR T. Given that CAR T follow-up care is less extended, rigorous, and complex than for stem cell transplantation, it may be more feasible to adequately support the underserved through and after CAR T, in order to level outcomes across race, ethnicity, and SES.

The strengths of this study include its large sample size given the comparative rarity of B-ALL, detailed clinical data regarding CAR T delivery, and our ability to include patients from a wide geographic area across the US. This broad patient base enhances the generalizability of these findings and ensures a more representative assessment of CAR T therapy’s effectiveness across varied demographic and clinical settings in the US, however, our findings may not be applicable in other countries with different socioeconomic structure and healthcare delivery infrastructure. There are several limitations with our study. Firstly, assessing SDI based on zip code alone may not accurately describe a patient’s SES or social situation, ultimately leading to misclassification bias. Social deprivation and vulnerability may be better estimated using more specific census tract data, combining it with the SDI as well as other indices. Importantly, this study did not capture patients for whom CAR T-cell therapy may otherwise have been indicated, but who ultimately did not get referred or make it to infusion. The reasons for failure to reach CAR T therapy are numerous, and barriers to access may be present even prior to CAR T referral [49]. The specific factors that impact access to CAR T will require further study in order to tackle barriers, ideally with a population of CAR T eligible patients as the denominator.

To our knowledge, this multicenter retrospective analysis is the first to assess racial, ethnic, and SDoH differences as they relate to the safety and outcomes of brexu-cel for R/R B-ALL outside of a clinical trial setting. We found no difference in PFS or OS in Hispanic patients and no difference in OS based on SDoH factors. Receiving CAR T therapy may eliminate some of the historical disparities in outcomes seen in patients with B-ALL. Ensuring equal access to these advanced therapies is critical to promoting equity in health outcomes for all patients with R/R B-ALL.

## Supplementary information

SUPPLEMENTAL MATERIAL

## Data Availability

Data from the current work are available on request.
