# Supplementary material for: The impact of social determinants of health on outcomes of brexucabtagene autoleucel in adults with relapsed/refractory B-cell acute lymphoblastic leukemia
Source: Bone Marrow Transplant. 2025 Aug 23;60(11):1465–71. doi: 10.1038/s41409-025-02693-0 (PMC12583187; doi:10.1038/s41409-025-02693-0)

**Supplement**

|  |  | OR | LCL | UCL | p-value |
| --- | --- | --- | --- | --- | --- |
| SDOH |  |  |  |  |  |
| Race/ethnicity | Hispanic | 0.06 | 0.00 | 0.86 | 0.96 |
|  | Black | 0.03 | 0.00 | 0.57 | 0.95 |
|  | Asian/Pacific Islander | 0.11 | 0.00 | 2.57 | 0.97 |
|  | Non-Hispanic white (reference) | -- | -- | -- | -- |
| Insurance status | Government/Public | 2.65 | 0.47 | 15.01 | 0.03 |
|  | Private (reference) | -- | -- | -- | -- |
| Distance to CART site | Less than 50 miles | 2.93 | 0.58 | 14.73 | 0.19 |
|  | 50 miles or greater (reference) | -- | -- | -- | -- |
| Social Deprivation Index | Low (0-25th percentile) | 0.24 | 0.02 | 2.58 | 0.26 |
|  | Low-medium (26-50th percentile) | 1.24 | 0.07 | 21.61 | 0.45 |
|  | Medium-high (51-75th percentile) | 0.38 | 0.05 | 3.07 | 0.57 |
|  | High (76-100th percentile) (reference) | -- | -- | -- | -- |
| Clinical status |  |  |  |  |  |
| Lines of therapy |  | 0.86 | 0.52 | 1.42 | 0.55 |
| Disease burden pre-apheresis | Active > 5% | 1.06 | 0.14 | 8.32 | 0.21 |
|  | MRD+/unknown | 10.26 | 0.51 | 206.32 | 0.08 |
|  | MRD- (reference) | -- | -- | -- | -- |

**Supplemental Table 1. Association of clinical characteristics and social determinants of health with any post-CAR-T response (CR/MRD-, CR/MRD+, or unknown) in a multivariate logistic regression model.** Abbreviations: CR, complete response; MRD-, measurable residual disease negative; MRD+, measurable residual disease positive; SDOH, social determinants of health; OR, odds ratio; LCL, lower control limit; UCL, upper control limit.

|  |  | Model estimates | | | |
| --- | --- | --- | --- | --- | --- |
|  |  | HR | LCL | UCL | p-value |
| SDOH |  |  |  |  |  |
| Race/ethnicity | Hispanic | 1.16 | 0.67 | 2.03 | 0.60 |
|  | Black | 1.72 | 0.72 | 4.11 | 0.22 |
|  | Other | 0.97 | 0.38 | 2.48 | 0.95 |
|  | Non-Hispanic white (reference) | -- | -- | -- | -- |
| Referral source | Academic institution | 0.80 | 0.47 | 1.37 | 0.42 |
|  | Other | 0.84 | 0.25 | 2.76 | 0.77 |
|  | Private/community (reference) | -- | -- | -- | -- |
| Insurance status | Private | 1.18 | 0.71 | 1.98 | 0.53 |
|  | Government/Public (reference) | -- | -- | -- | -- |
| Distance to CAR T site | Less than 50 miles | 1.69 | 0.96 | 2.97 | 0.07 |
|  | 50 miles or greater (reference) | -- | -- | -- | -- |
| Relocated for CAR T therapy | Yes | 0.91 | 0.53 | 1.58 | 0.74 |
|  | No (reference) | -- | -- | -- | -- |
| Social Deprivation Index | High (76-100th percentile) | 1.97 | 0.78 | 4.99 | 0.15 |
|  | Medium-high (51-75th percentile) | 1.77 | 0.62 | 5.11 | 0.29 |
|  | Low-medium (26-50th percentile) | 4.19 | 1.68 | 10.44 | 0.002 |
|  | Low (0-25th percentile) (reference) | -- | -- | -- | -- |

**Supplemental Table 2. Univariable model for overall survival associated with social determinants of health**. Abbreviations: CAR T, chimeric antigen receptor T-cell; SDOH, social determinants of health; HR, hazard ratio; LCL, lower control limit; UCL, upper control limit.

|  |  | HR (95% CI) | p-value |
| --- | --- | --- | --- |
| SDOH |  |  |  |
| Race/ethnicity | Hispanic | 1.43 (0.56-3.65) | 0.45 |
|  | Black | 3.48 (1.01-12.03) | 0.048 |
|  | Asian/Pacific Islander | 1.62 (0.34-7.72) | 0.54 |
|  | Non-Hispanic white (reference) | -- | -- |
| Insurance status | Government/Public | 0.83 (0.40-1.74) | 0.63 |
|  | Private (reference) | -- | -- |
| Distance to CAR T site | Less than 50 miles | 1.92 (0.79-4.67) | 0.15 |
|  | 50 miles or greater (reference) | -- | -- |
| Social Deprivation Index | Low (0-25th percentile) | 0.24 (0.05-1.22) | 0.09 |
|  | Low-medium (26-50th percentile) | 2.03 (0.81-5.07) | 0.13 |
|  | Medium-high (51-75th percentile) | 0.78 (0.28-2.21) | 0.64 |
|  | High (76-100th percentile) (reference) | -- | -- |

**Supplemental Table 3. Multivariable Cox regression analysis of association of social determinants of health with overall survival.** Abbreviations: SDOH, social determinants of health; CAR T, chimeric antigen receptor T-cell; HR, hazard ratio; CI, confidence interval.

|  |  | Model estimates | | | |
| --- | --- | --- | --- | --- | --- |
|  |  | HR | LCL | UCL | p-value |
| SDOH |  |  |  |  |  |
| Race/ethnicity | Hispanic | 1.03 | 0.50 | 2.10 | 0.94 |
|  | Black | 1.90 | 0.73 | 4.94 | 0.19 |
|  | Asian/Pacific Islander | 1.61 | 0.51 | 5.10 | 0.42 |
|  | Non-Hispanic white (reference) | -- | -- | -- | -- |
| Insurance status | Government/Public | 1.01 | 0.56 | 1.82 | 0.98 |
|  | Private (reference) | -- | -- | -- | -- |
| Distance to CAR T site | Less than 50 miles | 1.37 | 0.72 | 2.60 | 0.34 |
|  | 50 miles or greater (reference) | -- | -- | -- | -- |
| Social Deprivation Index | Low (0-25th percentile) | 0.57 | 0.23 | 1.45 | 0.24 |
|  | Low-medium (26-50th percentile) | 1.24 | 0.59 | 2.62 | 0.58 |
|  | Medium-high (51-75th percentile) | 0.82 | 0.35 | 1.89 | 0.63 |
|  | High (76-100th percentile) (reference) | -- | -- | -- | -- |
| Clinical status |  |  |  |  |  |
| Lines of therapy |  | 0.94 | 0.78 | 1.12 | 0.47 |
| Disease burden pre-apheresis | Active > 5% | 1.92 | 0.87 | 4.21 | 0.10 |
|  | MRD+/unknown | 0.98 | 0.38 | 2.48 | 0.96 |
|  | MRD- (reference) | -- | -- | -- | -- |
| Consolidation/ maintenance | Transplant | 0.27 | 0.12 | 0.64 | 0.003 |
|  | Other | 0.24 | 0.07 | 0.81 | 0.02 |
|  | None (reference) | -- | -- | -- | -- |

**Supplemental Table 4.  Multivariable model for progression free survival associated with clinical characteristics and social determinants of health.** Abbreviations: SDOH, social determinants of health; HR, hazard ratio; LCL, lower control limit; UCL, upper control limit; CAR T, chimeric antigen receptor T-cell; MRD+, measurable residual disease positive; MRD-, measurable residual disease negative.

**Supplemental Figure 1a. Cumulative incidence of nonrelapse mortality by race, relapse as a competing risk.** Abbreviation: NHW, non-Hispanic white

**Supplemental Figure 1b. Cumulative incidence of nonrelapse mortality by social deprivation index, relapse as a competing risk**

**Figure 1a.**
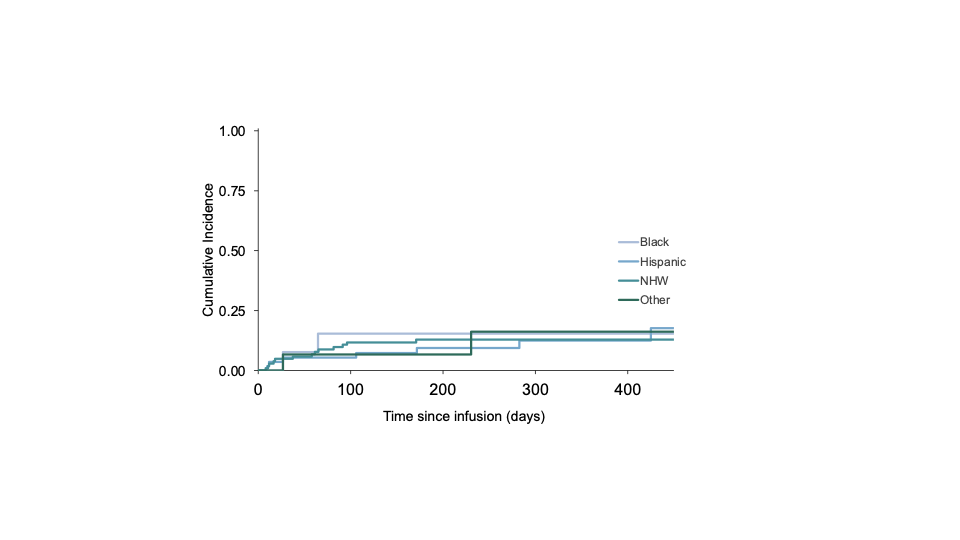
  
**Figure 1b.**
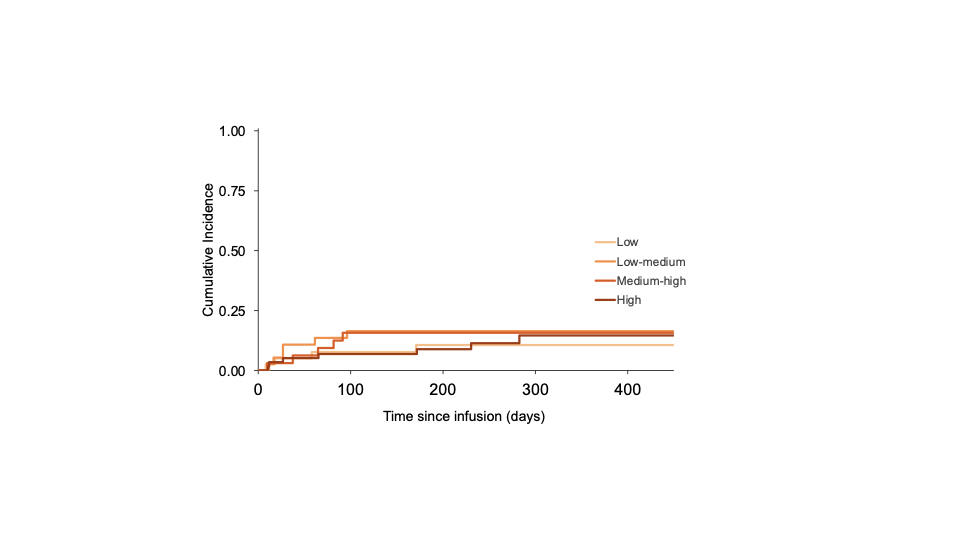

Supplement: Supplementary file 1 — SUPPLEMENTAL MATERIAL [file 41409_2025_2693_MOESM1_ESM.docx]
